# Stimulated acoustic emissions from coupled strings

**DOI:** 10.1007/s10665-013-9635-8

**Published:** 2013-07-13

**Authors:** Richard S. Chadwick, Jessica S. Lamb, Daphne Manoussaki

**Affiliations:** 1 Section on Auditory Mechanics, NIDCD—National Institute on Deafness and Other Communication Disorders, Bethesda, MD USA; 2 Division of Applied Mathematics, Department of Sciences, Technical University of Crete, Hania, Crete Greece

**Keywords:** Acoustic emissions, Asymptotic matching of inner and outer expansions, Coupled wave propagation, Mode conversion, WKB approximation

## Abstract

We consider traveling transverse waves on two identical uniform taut strings that are elastically coupled through springs that gradually decrease their stiffness over a region of finite length. The wave system can be decomposed into two modes: an in-phase mode ($+$) that is transparent to the coupling springs, and an out-of-phase mode ($-$) that engages the coupling springs and can resonate at a particular location depending on the excitation frequency. The system exhibits linear mode conversion whereby an incoming ($+$) wave is reflected back from the resonance location both as a propagating ($+$) wave and an evanescent ($-$) wave, while both types emerge as propagating forward through the resonance location. We match a local transition layer expansion to the WKB expansion to obtain estimates of the reflection and transmission coefficients. The reflected waves may be an analog for stimulated emissions from the ear.

## Introduction

Linear mode conversion is a phenomenon that has been studied for several decades and finds application in plasma physics, geophysics, and biophysics [1–3]. The slowly varying WKB approximation becomes singular at such a mode conversion point. Here we examine a simple mechanical system that illustrates that effect. Typically, one propagating wave can excite another at a location where the wavelengths of the different modes become similar and energy can be exchanged, although here an internal resonator excites the second mode. Of particular interest is to understand how a reflection can occur when there are no discontinuities in physical properties. Originally, Rayleigh [4] found reflections on a single string having smoothly varying mass over a finite transition region. Here we find reflections on two coupled strings with no discontinuities in physical properties. Our motivation is to find a simple model for stimulated emissions from the ear, a subclass of emissions found in the ear that occur at the frequency of the incoming stimulus.

## Coupled strings model

We consider the following coupled system:

1 $$\begin{aligned} \rho \frac{\partial ^{2}y_1 }{\partial t^{2}}&= T\frac{\partial ^{2}y_1 }{\partial x^{2}}-K(x)(y_1 -y_2) ,\\ \rho \frac{\partial ^{2}y_2 }{\partial t^{2}}&= T\frac{\partial ^{2}y_2 }{\partial x^{2}}+K(x)(y_1 -y_2), \nonumber \end{aligned}$$

where ($y _{1}, y_{2}$) are the transverse displacements, $\rho$ is the density, $T$ is the tension, and $K(x)$ is the variable stiffness of the coupling spring, which is assumed to exert a restoring force proportional to the difference in displacements of the two strings. It is easy to see that the system admits two uncoupled modes (+, $-$). The (+) mode does not engage the coupling spring and is governed by the homogenous wave equation

2 $$\begin{aligned} \rho \frac{\partial ^{2}y^{+}}{\partial t^{2}}=T\frac{\partial ^{2}y^{+}}{\partial x^{2}}. \end{aligned}$$

This equation can be obtained by adding the two equations in (1) and defining $y^{+}= y_{1} + y_{2}$. The ($-$) mode is governed by the inhomogeneous telegraph equation

3 $$\begin{aligned} \rho \frac{\partial ^{2}y^{-}}{\partial t^{2}}=T\frac{\partial ^{2}y^{-}}{\partial x^{2}}-2K(x)y^{-}. \end{aligned}$$

This equation can be obtained by subtracting the second equation in (1) from the first and defining $y^{-}= y_{1}-y_{2}$. It then follows that $y_{1}=(y^{+}+y^{-})/2$ and $y_{2}=(y^{+}- y^{-})/2$. These latter relations allow us to transform from modal coordinates back to physical coordinates. It is easiest, however, to solve a specific problem in modal coordinates. For simple harmonic motion with the stimulus frequency $\omega ,{\partial }/{\partial t}\rightarrow \mathrm{i}\omega$, and we consider the reduced uncoupled system:

4 $$\begin{aligned}&\frac{{\text{ d }}^{2}y^{+}}{{\text{ d }}\xi ^{2}}+y^{+}=0, \nonumber \\&\frac{{\text{ d }}^{2}y^{-}}{{\text{ d }}\xi ^{2}}+[1-\sigma (\xi )]y^{-}=0, \end{aligned}$$

where we have introduced the scaled quantities $\xi = kx, k = \omega /c, c^{2}=T/\rho$, and $\sigma = 2K/(\rho \omega ^{2})$. Consider exciting a right-running (+) wave mode of unit amplitude from a source far to the left $\xi \rightarrow -\infty$, with a $\pi /2$ phase lead over all other waves generated by a resonator (see below) at $\xi =0$. The coupling spring is set up such that to the left of the transition, the ($-$) mode cannot propagate. The general solution of system (4) can be expressed as a linear combination of the two solutions of each of the equations, totaling four in number. The first has the two solutions $y^{+} =\text{ exp }(- \mathrm{i}\xi )$ and $\text{ exp }(\mathrm{i}\xi )$, the first being an incoming right-running wave, and the second a reflected wave. Incoming left-running waves entering from the right $\xi \rightarrow \infty$ shall not be allowed.

## WKB solution away from turning point

Approximate right- and left-running WKB [5] solutions for the second equation in (4) are given by (5):

5 $$\begin{aligned} y^{-}=\frac{\exp \left( \mp \mathrm{i}\int {\sqrt{1-\sigma (\xi )} \,\,{\text{ d }}\xi } \right) }{\root 4 \of {1-\sigma (\xi )}}. \end{aligned}$$

The WKB approximation clearly fails at the turning point, where $\sigma =1$, and mode conversion can occur, possibly shifting energy to the ($-$) mode. Physically, a resonance occurs where $\sigma =1$, with $\omega =\sqrt{2K/\rho }$, i.e., the mass/length of the strings and the coupling spring form a locally resonant system. The factor of 2 arises because of a node at the center of the spring for the ($-$) mode, resulting in two springs in series, each having half the length and thus twice the stiffness. The singularity at $\sigma =1$ is due to the failure of the WKB energy equation. Our first goal is to salvage the WKB approximation, i.e., the outer expansion, by asymptotic matching to a local transition layer expansion, i.e., the inner expansion, constructed to be valid as $\xi \rightarrow 0$. The idea is to push the two expansions into their respective domains of invalidity to see if there is a region where both expansions agree. Here we show the inner and outer expansions indeed match to lowest order with this simplest form of matching. Langer [5] provided more accurate uniformly valid expansions for problems of this type. We proceed to calculate the reflection and transmission coefficients for the wave system.

## Transition layer (Airy function solutions)

The transition layer equation is obtained by considering a Taylor series for the function $\sigma (\xi )$ about the turning point where $\sigma =1$. To be specific, let us consider a piecewise continuous quadratic stiffness function with continuous first derivative that is antisymmetric about $\xi = 0$, which is the center of the transition from high uniform stiffness to low uniform stiffness defined by the domain $-\zeta /2\le \xi \le \zeta /2$. Let $1-\sigma (\xi ) = G(\xi ) =4\delta \xi /\zeta$ $(1+\xi /\zeta )$ for $-\zeta /2\le \xi \le 0$, and let $1-\sigma (\xi ) = F(\xi )=4\delta \xi /\zeta$ ($1-\xi /\zeta )$ for $0\le \xi \le \zeta /2$, where the total stiffness decrease is $2\delta$. Note that for $\xi <-\zeta /2, 1-\sigma (\xi ) = G(-\zeta /2)<0$, which implies that the ($-$) mode is evanescent on the left side of the transition, while for $\xi > \zeta /2, 1-\sigma (\xi ) =F(\zeta /2)>0$, which implies that the ($-$) mode can propagate on the right side of the transition. Keeping just the linear term, we consider the first-order transition equation

6 $$\begin{aligned} \frac{{\text{ d }}^{2}Y}{{\text{ d }}\xi ^{2}}+\gamma \xi Y=0, \end{aligned}$$

where $\gamma = 4\delta /\zeta >0$, which has a general solution in terms of the Airy functions

7 $$\begin{aligned} Y(\xi )=\alpha \mathrm{Ai}(-\root 3 \of {\gamma }\xi )+\beta \mathrm{Bi}(-\root 3 \of {\gamma }\xi ), \end{aligned}$$

where ($\alpha , \beta$) are coefficients to be determined. We shall require the leading terms of the asymptotic forms of the Airy functions for large values of the argument:

8 $$\begin{aligned}&\mathrm{Ai}(-\root 3 \of {\gamma }\xi )=\frac{\exp \left( -\frac{2}{3}\sqrt{\gamma }(-\xi )^{3/2}\right) }{2\sqrt{\pi }\root 12 \of {\gamma }\root 4 \of {-\xi }}+\cdots \end{aligned}$$

9 $$\begin{aligned}&\mathrm{Bi}(-\root 3 \of {\gamma }\xi )=\frac{\exp \left( \frac{2}{3}\sqrt{\gamma }(-\xi )^{3/2}\right) }{\sqrt{\pi }\root 12 \of {\gamma }\root 4 \of {-\xi }}+\cdots \end{aligned}$$

as $\xi \rightarrow -\infty$, while for $\xi \rightarrow \infty$ we have

10 $$\begin{aligned} \mathrm{Ai}(-\root 3 \of {\gamma }\xi )=\frac{\sin \left( \frac{2}{3}\sqrt{\gamma }\xi ^{3/2}+\frac{\pi }{4}\right) }{\sqrt{\pi }\root 12 \of {\gamma }\root 4 \of {\xi }}+\cdots \,, \end{aligned}$$

11 $$\begin{aligned} \mathrm{Bi}(-\root 3 \of {\gamma }\xi )=\frac{\cos \left( \frac{2}{3}\sqrt{\gamma }\xi ^{3/2}+\frac{\pi }{4}\right) }{\sqrt{\pi }\root 12 \of {\gamma }\root 4 \of {\xi }}+\cdots . \end{aligned}$$

Since Eq. (9) becomes unbounded as $\xi \rightarrow -\infty$, we must reject the Bi function to the left of the turning point, while Eq. (11) shows we can keep it to the right of the turning point.

Equations (8) and (9) suggest that to the left of the turning point we write the WKB solution in the form

12 $$\begin{aligned} y^{-}(\xi )=A_\mathrm{{l}} \frac{\exp \left( -\int _0^{-\xi } {\sqrt{G(\tau )}}\,\,{\text{ d }}\tau \right) }{\root 4 \of {G(\xi )}}+B_\mathrm{{l}} \frac{\exp \left( \int _0^{-\xi } {\sqrt{G(\tau )}}\,\,{\text{ d }}\tau \right) }{\root 4 \of {G(\xi )}}. \end{aligned}$$

Expanding (12) as $\xi \rightarrow 0$ gives

13 $$\begin{aligned} y^{-}(\xi )=A_\mathrm{{l}} \frac{\exp \left( -2/3\sqrt{\gamma }(-\xi )^{3/2}\right) }{\root 4 \of {\gamma \xi }}+B_\mathrm{{l}} \frac{\exp \left( 2/3\sqrt{\gamma }(-\xi )^{3/2}\right) }{\root 4 \of {\gamma \xi }}+\cdots . \end{aligned}$$

Expressions (7), (8), and (13) asymptotically match for $\xi < 0$ provided

14 $$\begin{aligned} A_\mathrm{{l}}&= \frac{1}{2\sqrt{\mathrm{i}\pi }}\root 6 \of {\gamma }\alpha , \end{aligned}$$

15 $$\begin{aligned} B_\mathrm{{l}}&= 0. \end{aligned}$$

Similarly, Eqs. (10) and (11) suggest that to the right of the turning point we write the WKB solution in the form

16 $$\begin{aligned} y^{-}(\xi )=A_\mathrm{{r}} \frac{\sin \left( \int _0^\xi {\sqrt{F(\tau )}}\,\,{\text{ d }}\tau +\pi /4 \right) }{\root 4 \of {F(\xi )}}+B_\mathrm{{r}} \frac{\cos \left( \int _0^\xi {\sqrt{F(\tau )}}\,\,{\text{ d }}\tau +\pi /4\right) }{\root 4 \of {F(\xi )}}. \end{aligned}$$

Expanding (10) as $\xi \rightarrow 0$ gives

17 $$\begin{aligned} y^{-}(\xi )=A_\mathrm{{r}} \frac{\sin \left( 2/3\sqrt{\gamma }\xi ^{3/2}+\pi /4\right) }{\root 4 \of {\gamma \xi }}+B_\mathrm{{r}} \frac{\cos \left( 2/3\sqrt{\gamma }\xi ^{3/2}+\pi /4\right) }{\root 4 \of {\gamma \xi }}+\cdots . \end{aligned}$$

Expressions (7), (10), (11), and (13) asymptotically match for $\xi > 0$ provided

18 $$\begin{aligned} A_\mathrm{{r}} =\frac{1}{\sqrt{\pi }}\root 6 \of {\gamma }\alpha , \end{aligned}$$

19 $$\begin{aligned} B_\mathrm{{r}} =\frac{1}{\sqrt{\pi }}\root 6 \of {\gamma }\beta . \end{aligned}$$

## Interface conditions

We can now proceed to calculate the complex amplitude coefficients ($R^{+}, R^{-}, T^{+}, T^{-}, \alpha , \beta$) of all the waves resulting from the interaction with the local resonator. $R^{+}$ and $R^{-}$ are reflection coefficients that denote the amplitudes of the reflected left-running ($+$) and ($-$) wave modes that result from the interaction of the right-running ($+$) wave of unit amplitude with a smooth decrease in coupling spring stiffness. Similarly, $T^{+}$ and $T^{-}$ (not to be confused with the tension $T$ in the strings) are the transmission coefficients that denote the amplitudes of the right-running ($+$) and ($-$) wave modes transmitted through the transition. To do this we select three locations $\xi =-\zeta /2,0, \zeta /2$ and require that the amplitude $A$ and slope $S$ of the total wave system be continuous at these locations, corresponding to unbroken and unplucked strings. This results in a system of six linear equations for the six unknown amplitudes. We note that if $A$ and $S$ are separately continuous, then their product *AS*, the wave energy, is also continuous. Thus our scheme conserves the total energy. Nevertheless, the ($+$) and ($-$) modes can still exchange energy, as we will see.

To establish the equations at each interface, we sum the product of the wave amplitude and phase for right-running and left-running waves for both ($+$) and ($-$) modes. We choose the wave phases to be zero at the turning point $\xi =0$, which assumes that new waves generated there lag the right-running (+) source wave by $\pi /2$, consistent with a phase lag at resonance. First we note that for $\xi$ $<-\zeta /2$, we have exp $(-\mathrm{i}(\xi -\pi /2))$ and $R^{+} \text{ exp } (\mathrm{i}\xi )$ as the right- and left-running solutions, respectively, of the first of Eq. (4), while $R^{-}\exp (\sqrt{\delta }\xi )$ is the acceptable (evanescent) solution of the second of Eq. (4). Similarly, we have for $\xi$ >$\zeta /2, T^{+}\text{ exp } (-\mathrm{i}(\xi -\pi /2))$ and $T^{-}\exp (-\mathrm{i}\sqrt{\delta }\xi )$ as acceptable (right-running) solutions of Eq. (4). Furthermore, inside the transition region, $-\zeta /2< \xi < \zeta /2, \text{ exp } (-\mathrm{i}(\xi -\pi /2))$ and $R^{+} \text{ exp } (\mathrm{i}\xi )$ are still allowable solutions of the first of Eq. (4), whereas the solutions of the second of Eq. (4) are approximated by the WKB solutions. Just inside $\xi =\zeta /2$, we use Eqs. (16), (18), and (19) to obtain the wave amplitudes

$$\begin{aligned} \frac{\root 6 \of {\gamma }}{\sqrt{\mathrm{i}\pi }\root 4 \of {\delta }}\left[ \alpha \cos \left( \frac{\pi \zeta \sqrt{\delta }}{8}+\frac{\pi }{4}\right) +\beta \sin \left( \frac{\pi \zeta \sqrt{\delta }}{8}+\frac{\pi }{4}\right) \right] . \end{aligned}$$

Similarly, just to the right of $\xi =- \zeta /2$, we use Eqs. (12), (14), and (15) to obtain

$$\begin{aligned} \frac{\root 6 \of {\gamma }}{\sqrt{\mathrm{i}\pi }\root 4 \of {\delta }}\frac{\alpha }{2}\exp \left( -\frac{\pi \zeta \sqrt{\delta }}{8}\right) . \end{aligned}$$

Near the origin $\xi = 0$, we use the Airy function solution Eq. (7), instead of the WKB approximation, but we reject the Bi function to the left of $\xi =0$ because of exponential growth. It seems necessary to have a discontinuous solution of the ($-$) mode near the origin to get a nontrivial solution of the mode conversion system. This is physically permissible, however, since we still maintain continuity of the sum of the ($+$) and ($-$) modes at the origin.

Keeping all this in mind, the interface conditions at $\xi = - \zeta /2$ are

20 $$\begin{aligned}&\exp (\mathrm{i}[\zeta /2+\pi /2])+R^{+}\exp (-\mathrm{i}\zeta /2)+R^{-}\exp \left( -\zeta \sqrt{\delta }/2\right) =\frac{\root 6 \of {\gamma }}{\sqrt{\mathrm{i}\pi }\root 4 \of {\delta }}\frac{\alpha }{2}\exp \left( -\frac{\pi \zeta \sqrt{\delta }}{8}\right) , \end{aligned}$$

21 $$\begin{aligned}&-\mathrm{i}[\exp (\mathrm{i}[\zeta /2+\pi /2])-R^{+}\exp (-\mathrm{i}\zeta /2)]+\sqrt{\delta }R^{-}\exp \left( -\zeta \sqrt{\delta }/2\right) =-\frac{\root 4 \of {\delta }\root 6 \of {\gamma }}{\sqrt{\mathrm{i}\pi }}\frac{\alpha }{2}\exp \left( -\frac{\pi \zeta \sqrt{\delta }}{8}\right) . \end{aligned}$$

Equation (20) stipulates continuity of amplitude $A$, while Eq. (21) stipulates continuity of the wave slope $S$, to the first approximation. The first term on the left represents the incoming ($+$) mode wave having an amplitude assumed to be unity, corresponding to the physical displacements $y_{1} = y_{2} = 1/2$. This is the only external forcing in the problem. All other waves are a result of the interaction of this wave with the internal resonator at $\xi =0$. The second term is the reflected (+) mode wave, and the third term is the reflected ($-$) mode wave, which is evanescent since the phase is imaginary and results in an exponential decay. Note that there is no incoming ($-$) evanescent mode wave since it would have already decayed had it been excited. The terms on the right represent the WKB approximation to these waves across the interface. The interface continuity conditions at $\xi =0$ are

22 $$\begin{aligned}&\exp (\mathrm{i}\pi /2)+R^{+}=T^{+}\exp (\mathrm{i}\pi /2)+\beta \mathrm{Bi}(0), \end{aligned}$$

23 $$\begin{aligned}&-\mathrm{i}[\exp (\mathrm{i}\pi /2)-R^{+}]=-\root 3 \of {\gamma }\beta \mathrm{Bi}^{\prime }(0)-\mathrm{i}T^{+}\exp (\mathrm{i}\pi /2). \end{aligned}$$

Note that the Ai function is continuous across the interface and thus cancels. If Bi were continuous and also canceled, we would get the unwanted (unless $\delta = 0$) trivial solution $T^{+} =1$ and $R^{+} =0$. Interestingly, the solution is evidently not unique at $\xi =0$ according to the demonstration that follows. Consider the first Eq. (1), and make the following substitutions: $\partial /\partial t\rightarrow \mathrm{i}\omega , \partial /\partial x\rightarrow -\mathrm{i}k, K(0)\rightarrow \rho \omega ^{2}/2$. The last substitution is the resonance condition. The following dispersion relation then results:

$$\begin{aligned} 1-2\frac{k^{2}T}{\rho \omega ^{2}}+\frac{y_2 }{y_1 }=0. \end{aligned}$$

This relationship shows there is a continuum of wavenumbers that can exist that depend on the mode ratio $y_{2}/y_{1}$. Furthermore, this relation shows that the ($-$) mode is excitable at $\xi =0$. If we suppose $y_2 /y_1 \rightarrow -(1+\vartheta )$, where $0<\vartheta \ll 1$ is a small perturbation that could either be thermal or the result of an evanescent incoming right-running wave, then it follows that the wave speed of the perturbation $c=\sqrt{\omega /k}=\sqrt{-2T/(\rho \vartheta )}$. The large imaginary wave speed is indicative of the excitability of the ($-$) mode due to the local resonance condition. Thus we are entitled to seek a nontrivial solution containing the ($-$) mode as long as the required continuity properties are satisfied.

Finally, the interface conditions at $\xi =\zeta /2$ are

24 $$\begin{aligned}&\frac{\root 6 \of {\gamma }}{\sqrt{\mathrm{i}\pi }\root 4 \of {\delta }}\left[ \alpha \cos \left( \frac{\pi \zeta \sqrt{\delta }}{8}\!+\!\frac{\pi }{4}\right) +\beta \sin \left( \frac{\pi \zeta \sqrt{\delta }}{8}\!+\!\frac{\pi }{4}\right) \right] \!=\!T^{+}\exp \left( -\mathrm{i}\zeta /2+\mathrm{i}\pi /2\right) +T^{-}\exp \left( -\mathrm{i}\zeta \sqrt{\delta }/2\right) , \end{aligned}$$

25 $$\begin{aligned}&\frac{\root 4 \of {\delta }\root 6 \of {\gamma }}{\sqrt{\mathrm{i}\pi }}\left[ \!-\alpha \sin \left( \frac{\pi \zeta \sqrt{\delta }}{8}\!+\!\frac{\pi }{4}\right) \!+\!\beta \cos \left( \frac{\pi \zeta \sqrt{\delta }}{8}\!+\!\frac{\pi }{4}\right) \right] \!=\!-\mathrm{i}\left[ T^{\!+\!}\exp \left( -\mathrm{i}\zeta /2\!+\!\mathrm{i}\pi /2\right) +\sqrt{\delta }T^{-}\exp \left( \!-\mathrm{i}\zeta \sqrt{\delta }/2\right) \right] .\nonumber \\ \end{aligned}$$

The amount of energy converted in the process can be calculated in terms of the reflection and transmission coefficients. The total incoming wave energy is unity, all of it being in the (+) mode. The amount of energy in the ($-$) mode leaving the transition boundaries is

26 $$\begin{aligned} \sqrt{\delta }\left\{ -\mathrm{i}(T^{-})^{2}\exp \left( -\mathrm{i}\zeta \sqrt{\delta }\right) +(R^{-})^{2}\exp \left( -\zeta \sqrt{\delta }\right) \right\} . \end{aligned}$$

The first term represents energy from a wave that leaves outward from the right interface while the second is evanescent and leaves through the left interface.

## Results and discussion

**Fig. 1:**
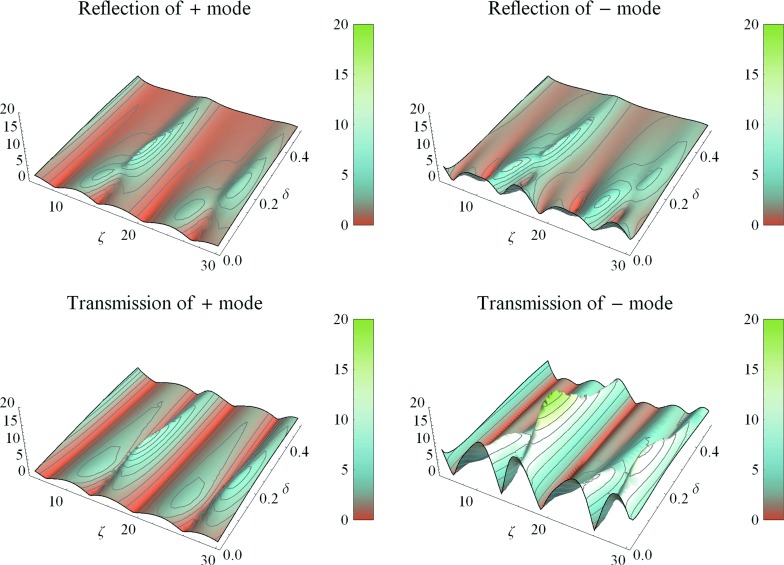
Computed reflection and transmission coefficients. *Color* is coded to absolute values of $R^{+}$ and $R^-\exp \left( -\zeta \sqrt{\delta }/2\right)$ (*upper panel*) and $T^{+}$ and $T^{-}$(*lower panel*). The scaled length and height parameters of the stiffness transition, $\zeta$ and $\delta$, respectively, are the other axes in these plots

The amplitudes of the complex reflection and transmission coefficients are plotted in Fig. 1 as a function of the two parameters $\delta$ and $\zeta$. The difference between high and low values of the coupling spring stiffness is $2\delta$, and $\zeta /(2\pi )$ is the ratio of the length of the transition to the wavelength of the incoming (+) wave. Thus, when $\zeta = 2\pi$, one incoming wavelength just fits into the confines of the transition region. All amplitudes have a nonmonotonic and complex dependence with respect to both parameters. The accuracy of the correction to the WKB approximation near a mode conversion point remains an interesting question that needs to be addressed in future work. In particular, limitations on the smallness of the stiffness decrease need to be established. Also, the question of how to deal with an isolated turning point, which is not enclosed by well-defined interfaces, remains unanswered by the present analysis.

The oscillatory behavior of the reflection coefficients with respect to $\zeta$ shows that even this simple system exhibits a stimulated emission spectrum that is characteristic of the ear. In the present system, the frequency spacing of the spectrum originates from the oscillatory nature of the Airy functions, given by Eqs. (10) and (11). Physically, it is due to the coupling stiffness gradient, and a local resonance between the coupling spring and the mass of the strings. In the mammalian ear there are numerous contributions to a decreasing coupling stiffness gradient in the organ of Corti. The increasing length of outer hair cells and their stereocilia from base to apex are an example. In contrast, the coherent reflection theory that Zweig and Shera [6] developed for the ear argues that the incoming wave is scattered by local irregularities of any kind and then coherently filtered by the incoming wave.
